# The reliability and validity of the Swedish translation of the Vertigo Symptom Scale – short form in a cohort with acute vestibular syndrome

**DOI:** 10.1080/07853890.2025.2457517

**Published:** 2025-02-10

**Authors:** Solmaz Surano, Erik Faergemann, Gabriel Granåsen, Jonatan Salzer

**Affiliations:** a Department of Clinical Sciences, Neurosciences, Umeå University, Umeå, Sweden; b Department of Public Health and Clinical Medicine, Umeå University, Umeå, Sweden

**Keywords:** Vertigo symptom scale short form, Swedish translation, acute vestibular syndrome, vestibular rehabilitation, vertigo, dizziness, psychometric properties, reliability, validity

## Abstract

**Background:**

The Vertigo Symptom Scale – short form (VSS–SF) is commonly used to measure dizziness and vertigo over the past month. This study aimed to (1) adapt the VSS–SF for the Swedish population and assess its psychometric properties, and (2) develop a modified version for measuring symptoms in the acute phase of acute vestibular syndrome (AVS).

**Methods:**

The VSS–SF was translated into Swedish and adapted cross-culturally. Its psychometric properties were evaluated in 86 AVS patients, both in the acute stage (1–7 days from symptom onset) with a modified acute version, and after six weeks of vestibular rehabilitation using the standard VSS–SF. Factor structure, convergent and discriminant validity, and internal consistency were analyzed. Test-retest reliability was assessed at six weeks. Participants were also evaluated with the Dizziness Handicap Inventory (DHI) and balance tests. Controls included 54 healthy participants.

**Results:**

Exploratory factor analysis revealed a two-factor structure for both versions, corresponding to vertigo-balance (VSS–V) and autonomic-anxiety (VSS–A) subscales. Both versions demonstrated strong factor structures with adequate loadings. Internal consistency was high for the standard version (Cronbach’s alpha 0.76 to 0.87) and for the total and VSS–V subscale of the acute version (0.82 and 0.85, respectively), but poor for the acute VSS–A subscale (0.50). Convergent validity was supported by Spearman’s rank correlations. The discriminative ability was excellent for the acute VSS–SF and VSS–V (AUC 0.98 and 0.99), and acceptable for VSS–A (AUC 0.77). After six weeks, discriminative ability decreased but remained above 0.5. Test-retest reliability at six weeks was excellent for all scales (ICC 0.94, 0.93, and 0.93 for VSS–SF, VSS–V, and VSS–A).

**Conclusions:**

The VSS–SF was successfully adapted for the Swedish population, including an acute version for early dizziness assessment. Both versions confirmed a robust two-factor structure, with the acute version showing excellent early discriminative ability, particularly for the vertigo-balance dimension. However, the autonomic-anxiety subscale showed weaker psychometric properties, suggesting limited suitability for AVS patients. The adapted scales show promise for clinical use in diagnosing and evaluating dizziness and vertigo in the Swedish population.

**Trial registration:**

Clinicaltrials.gov Identifier NCT05056324, September 24, 2021. https://clinicaltrials.gov/ct2/show/NCT05056324

## Background

Dizziness and vertigo are commonly reported symptoms in patients seeking medical care, often leading to multiple emergency department visits. Estimates of the lifetime prevalence of significant dizziness range from 17 to 30% [1]. A population-based study found a 7.4% lifetime prevalence of vertigo caused by vestibular disease [2]. The International Classification of Vestibular Disorders system distinguishes vertigo from dizziness as separate conditions: dizziness as a distortion of spatial orientation without motion, and vertigo as the false perception of motion in any direction [3]. Nevertheless, the subjective and diverse manifestations of dizziness and vertigo can pose clinical challenges, as the symptoms are typically vague and difficult for patients to describe [4] and can be challenging for healthcare providers to diagnose due to the complexity and variety of the underlying causes [5]. This complexity is heightened by the intertwining of psycho-physiological factors such as the link between anxiety and dizziness, which can further impact a patient’s quality of life and recovery [6]. The use of patient-reported scales that examine both vestibular-balance and psycho-physiological aspects are valuable tools for assessing the severity of symptoms and the efficacy of treatments like vestibular rehabilitation [7].

The Vertigo Symptom Scale (VSS), developed based on patient interviews and literature, assesses the frequency and severity of dizziness symptoms [8]. It includes the original long version with 36 items and a short form (VSS–SF). Both are designed to measure symptom frequency and severity but over different time frames. The long version measures symptom frequency over the past year, while the VSS–SF measures symptoms within the past month. The latter has been used for assessing therapeutic effects in clinical trials [7,9]. Although the VSS–SF can be used as a single measure, it was designed to yield two distinct subscales: one measuring the severity of vertigo, dizziness, and unsteadiness (VSS–V), and the other focusing on autonomic symptoms such as nausea, heart pounding, and sweating (VSS–A) [10].

The translation and adaptation of patient-reported scales into a different language can potentially affect their psychometric properties, necessitating careful validation and consistency checks in accordance with international guidelines for patient-reported health outcomes. The long version of the VSS has previously been translated and validated in several languages, including Spanish, Turkish, Persian, and German [11–14]. Although the VSS long version has also been translated into Swedish, its validity was not investigated [15]. The psychometric properties of the VSS–SF have been assessed during cross-cultural validation of the Norwegian and Japanese versions [16,17], and the scale has also been translated and validated in Kurdish [18]. These versions demonstrated satisfactory internal consistency, reliability, convergent and discriminating validity, indicating the VSS–SF’s applicability across diverse cultural contexts. In the Norwegian and Kurdish adaptation, two factors, VSS–V and VSS–A, were extracted, in line with the original intended subscales of the VSS–SF [10]. In the Japanese version, an additional factor concerning the duration of symptoms was also identified.

The use of the VSS–SF in the acute stage of vertigo is justified by its ability to capture both vertigo and autonomic symptoms in a time-sensitive manner, making it suitable for assessing acute vestibular conditions. The scale’s two subscales—vertigo-balance and autonomic-anxiety symptoms—allow for comprehensive symptom assessment, even within the narrow time window of acute symptom onset, providing clinicians with valuable information during early stages of intervention. The current study thus aimed to translate and cross-culturally adapt the VSS–SF to Swedish, ensuring its applicability and relevance to a Swedish population with acute vestibular syndrome (AVS) in both the acute stage (adapting the VSS–SF to assess symptoms within the past 24 h), and after six weeks of vestibular rehabilitation (using standard version of the VSS–SF). AVS is defined as the sudden onset of dizziness or vertigo, nausea or vomiting, head motion intolerance, gait instability, and frequently nystagmus lasting for at least 24 h [19,20]. Acute unilateral vestibulopathy, encompassing disorders such as vestibular neuritis or ischemic labyrinthopathy, is a common cause of AVS while in rare cases a stroke in the posterior circulation may explain the symptoms [20].

In the larger context, this study aims to provide healthcare professionals with a reliable and valid tool for evaluating vestibular disorders in a Swedish-speaking population. By ensuring that the VSS–SF is appropriately adapted for Swedish users, the study seeks to facilitate better assessment and management of vestibular symptoms, including the assessment of interventions, ultimately contributing to improved patient care for individuals suffering from these challenging conditions.

## Methods

### Participants

A cross-sectional, multicentre, psychometric evaluation was performed as part of a larger randomized, controlled, evaluator-blinded, multicentre superiority trial evaluating the effect of web-based vestibular rehabilitation after acute vertigo [21]. All study participants were evaluated for complaints of acute onset dizziness or vertigo from October 2021 to October 2023. They were recruited from neurology or otolaryngology departments of four university hospitals and five general hospitals (six in urban areas and three in more rural areas) in Sweden. Eligible participants were at least 18 years old, had given written consent to participate in the study, had pathological spontaneous or gaze-evoked nystagmus and were symptomatic at inclusion. Screening and inclusion were within 7 days of the onset of continuous symptoms. All participants were proficient Swedish speakers with sufficient knowledge of how to use a smartphone, tablet, or computer. Exclusion criteria included pre-existing vestibular or neurological diseases, inability to use the online rehabilitation tool, mental or language difficulties affecting study comprehension, medical or physical contraindications to required movements, and regular use of medications like anticonvulsants, antiemetics, benzodiazepines, and neuroleptics. Recurring AVS without prior diagnosis and transient treatments for current vertigo were permitted.

The participants were randomized to either vestibular rehabilitation through written instructions (control), or vestibular rehabilitation through an Internet-based Vestibular Rehabilitation tool, YrselTräning (intervention). Medical examination was supplemented with an extensive battery of tests including walking and postural balance tests and several questionnaires, including the VSS–SF and the Dizziness Handicap Inventory (DHI). The tests were performed both at inclusion (baseline testing) and at the conclusion of the 6-week rehabilitation period. The target for this sub-study validating the Swedish version of the VSS–SF were to include the first 100 participants who completed the 6-week vestibular rehabilitation. For the test-retest study, all participants completed the VSS–SF twice, 48 h apart, at the end of the 6-week rehabilitation period. The decision to assess test-retest reliability after six weeks rather than during the acute phase was based on the inherent variability of symptoms in the acute stage of AVS. During the acute phase, rapid fluctuations and improvements in symptoms can introduce variability that is unrelated to the scale’s reliability, potentially undermining the stability of the results. By conducting the test-retest after six weeks, we aimed to capture a more stable symptom profile, providing a more accurate assessment of the VSS-SF’s consistency over time. The 48-hour interval was considered appropriate in line with previous studies [16,17]. Although there are no specific guidelines for the length of the time interval in test-retest studies, 48 h is likely enough time to ensure that prior responses are forgotten and brief enough for the condition to remain stable [16]. The control group, 54 healthy participants with no history of vestibular diseases or dizziness, were recruited from a parallel study investigating balance evaluations using a mobile phone application.

All data was collected at each centre by a local study coordinator except for the VSS–SF at six weeks (including test-retest study), which was collected through phone interviews by a blinded study coordinator since this is the primary endpoint in the multicentre randomized controlled trial (RCT).

### Ethics

This study complies with the Declaration of Helsinki. Ethical approval of the study protocol was obtained from the Swedish Ethical Review Authority (Etikprövningsmyndigheten), Uppsala, Sweden on 7 April 2021 (study ID: CIV-21-05-036744). Ethical approval for the control group was received from the Swedish Ethical Review Authority, Uppsala, Sweden on 24 August 2022 (Dnr 2022-03735-01). All participants gave their written, informed consent to take part in the study. This study adhered to the Consolidated Standards of Reporting Trials (CONSORT) guidelines.

### Measurements

#### Vertigo symptom scale – short form (VSS–SF)

The VSS–SF, a shortened version of the 36-item Vertigo Symptom Scale (VSS), assesses the severity of vertigo symptoms experienced within the past month [10]. It comprises 15 selected items from the VSS [22]. Patients are asked to rate the frequency of these symptoms on a five-point Likert scale of 0 to 4 (0 indicating no symptoms and 4 signifying symptoms occurring most days), resulting in total scores ranging from 0 to 60. Higher scores signify more severe symptoms, with severe dizziness defined as ≥12 points on the total scale. *A* ≥ 3-point difference from the baseline score is considered clinically significant [23,24]. The VSS–SF has been suggested to comprise two subscales: the vertigo-balance sub-scale consisting of 8 items (VSS–V; score range 0–32), and the autonomic-anxiety sub-scale comprising seven items (VSS–A; score range 0–28).

#### Dizziness handicap inventory (DHI)

The Dizziness Handicap Inventory (DHI) is a self-assessment tool designed to quantify the impact of dizziness and vertigo on daily life, aiding clinicians in assessing symptom severity and treatment efficacy. This 25-item questionnaire is divided into three subscales: Functional (9 items, max 32 points), assessing daily activity disruption; Emotional (9 items, max 40 points), evaluating affective responses like anxiety and stress; and Physical (7 items, max 28 points), focusing on symptoms and triggers. Scores range from 0 to 100, with higher scores indicating more severe handicap: 16-34 points suggest a mild handicap, 36-52 points a moderate handicap, and 54 or more points a severe handicap. The original version [25] has been translated and validated into multiple languages, including the Swedish version [26] used in this study.

#### Balance test

The balance data were collected by having participants stand on a balance foam pad with their feet together and folding both arms across their chest for a maximum of 30 s or until fail (coming off balance by extending either arm out, taking a step on or off the foam pad, or about to fall). The time to fail or completion was recorded using a stopwatch.

#### Average timed 25-foot walk (T25-FW)

Participants were timed using a stopwatch, recording the mean of two, timed attempts at walking a clearly marked 25-foot course.

### Translation and cross-cultural adaptation

**Figure 1.**
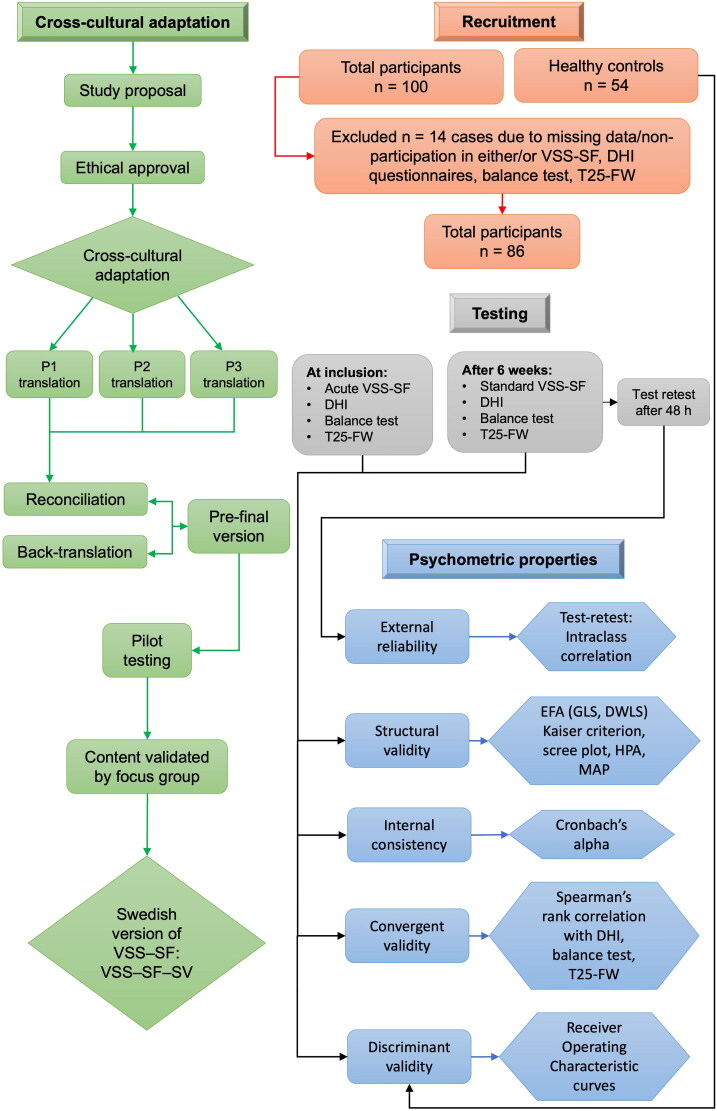
Flowchart of the study. Adapted from the flowchart by Zmnako and Chalabi [18]. The green and black arrows show the sequential order of the analyses. Abbreviations: P1/P2/P3 = person 1, person 2, and person 3 from the focus group. VSS–SF = vertigo symptom scale – short form, EFA = exploratory factor analysis, GLS = generalized least squares, DWLS = diagonally weighted least squares, HPA = horn’s parallel analysis, MAP = minimum average partial method, DHI = dizziness handicap inventory, T25-FW = timed 25-foot walk.

The VSS–SF was translated from the original English version into Swedish through a process of review, forward- and backward translations, modification, and cross-cultural adaptation. We followed established set of guidelines for the translation and cross-cultural adaptation of existing patient-reported outcome measures (PROMs) [27–29]. Permission was given by the creator of the scale, Lucy Yardley, to name it VSS–SF–SV (Swedish version), and it is presented together with the original English version in Table 1. The translation process consisted of separate translations in a focus group consisting of two physicians (a consultant neurologist and associate professor of neurology, and a junior physician and postdoctoral researcher in neuro-otology), and a physiotherapist in the neurology department involved in vertigo research. The individuals of the focus group are all familiar with dizzy patients, are native Swedish speakers, and fluent in English. Upon completion, the focus group met to compare the individual versions and agreed upon a merged version where concepts of clarity, fluency, and linguistic and cultural differences were taken into consideration. Some modifications were made in items 3, 6, 5, and 15 by deleting ‘feeling sick’ (item 3) and ‘swimmy’ (item 6 and 15) and using the Swedish word ‘hjärtklappning’ (item 5). Back-translation into English was performed by a senior researcher and associate professor in bilingualism, fluent in both Swedish and English, and who was blind to the original English version. Following back translation, the focus group compared the back translation to the original version to ensure semantic and conceptual equivalence and ultimately agreed upon a Swedish version of VSS–SF. Figure 1 provides a flowchart detailing the steps involved in the translation and cross-cultural adaptation process.

**Table 1.** The original English version and the translated Swedish version of the VSS–SF.

VSS–SF	VSS–SF, Swedish version
*How often in the past month have you had the following symptoms:*	*Hur ofta under den senaste månaden har du haft följande symtom:*
1. A feeling that either you, or things around you, are spinning or moving, lasting less than 20 min	1. En känsla av att du eller din omgivning snurrar eller rör på sig, känslan varar mindre än 20 minuter
2. Hot or cold spells	2. Värmevallning eller köldkänsla
3. Nausea (feeling sick), vomiting	3. Illamående, kräkning
4. A feeling that either you, or things around you, are spinning or moving, lasting more than 20 min	4. En känsla av att du eller din omgivning snurrar eller rör på sig, känslan varar mer än 20 minuter
5. Heart pounding or fluttering	5. Hjärtklappning
6. A feeling of being dizzy, disoriented or "swimmy", lasting all day	6. En känsla av yrsel eller förvirring, känslan varar hela dagen
7. Headache, or feeling of pressure in the head	7. Huvudvärk eller tryckkänsla i huvudet
8. Unable to stand or walk properly without support, veering or staggering to one side	8. Oförmåga att stå eller gå ordentligt utan stöd, vacklar åt sidan när du går
9. Difficulty breathing, been short of breath	9. Andningssvårighet, andfåddhet
10. Feeling unsteady, about to lose balance, lasting more than 20 min	10. En känsla av ostadighet, håller på att tappa balansen, känslan varar mer än 20 minuter
11. Excessive sweating	11. Svettas överdrivet mycket
12. Feeling faint, about to black out	12. Svimningskänsla, att det svartnar för ögonen
13. Feeling unsteady, about to lose balance, lasting less than 20 min	13. En känsla av ostadighet, håller på att tappa balansen, känslan varar mindre än 20 minuter
14. Pains in the heart or chest region	14. Bröstsmärta
15. A feeling of being dizzy, disoriented or "swimmy", lasting less than 20 min	15. En känsla av yrsel eller förvirring, känslan varar mindre än 20 minuter
**Response categories**	
0. Never 1. A few times 2. Several times 3. Quite often (every week) 4. Very often (most days)	0. Aldrig 1. Enstaka gånger 2. Flera gånger 3. Ganska ofta (varje vecka) 4. Väldigt ofta (nästan varje dag)
**VSS–V items:** 1, 3, 4, 6, 8, 10, 13, 15 **VSS–A items:** 2, 5, 7, 9, 11, 12, 14	

Another modified form of the VSS–SF was made in Swedish for baseline measurements in the acute stage of AVS. Here, the question ‘How often in the past month have you had the following symptoms is not applicable as symptom onset for the participants at baseline is between 24 h to 7 days prior to inclusion in the study in addition to having ongoing symptoms. Because of this, the question was revised to inquire about symptoms that have occurred during the past 24 h and as a result, the response categories were adjusted accordingly (Table 2). This modified scale is hereafter referred to as the VSS–SF acute version.

**Table 2.** The modified question and response categories for the VSS–SF, acute version.

VSS–SF, acute version	VSS–SF–SV, acute version
*How often in the past 24 h have you had the following symptoms?*	*Hur ofta under det senaste dygnet har du haft följande symtom?*
0. Never 1. A few times 2. Several times 3. Quite often (almost every hour) 4. Very often (almost always)	0. Aldrig 1. Enstaka gånger 2. Flera gånger 3. Ganska ofta (nästan varje timme) 4. Väldigt ofta (nästan hela tiden)

Four patients with acute dizziness who were either native or proficient Swedish speakers underwent pilot testing with the Swedish version of VSS–SF as well as the VSS–SF acute version to further examine its cross-cultural adaptability before conclusively defining the final Swedish version of the VSS–SF.

### Statistical analysis

#### Data screening

The demographic and test data were evaluated using descriptive statistical techniques. Cases with missing data were excluded. Floor or ceiling effects were considered significant when more than 15% of participants achieved the lowest or highest possible scores, respectively. The presence of these effects suggests a lack of extreme items at either end of the scale, indicating a potential shortfall in content validity [30,31]. The normality of each element within the VSS–SF was assessed by analyzing histograms for individual items, subscales, and total scales. Additionally, score distributions were examined using quantile-quantile plots and by comparing the mean and median of the scale and its subscales. For our sample size (50 < *n* < 300), absolute Z-scores exceeding 3.29 indicated a non-normal distribution [32]. Ordinal data, such as responses from Likert scales, typically do not adhere to a normal distribution. Consequently, this deviation necessitates the use of nonparametric methods, which were employed in the current study [33].

### Construct validity determination

#### Structural validity

We conducted a confirmatory factor analysis (CFA) based on a two-factor model corresponding to the VSS–V and VSS–A subscales. Given that our data consists of ordinal variables, we used the diagonally weighted least squares (DWLS) method. Model fit was evaluated using the standardized root mean square residual (SRMR), comparative fit index (CFI), and root mean square error of approximation (RMSEA). According to guidelines, good fit is indicated by an SRMR of 0.08 or below, a CFI of 0.95 or above, and an RMSEA of 0.06 or below [34]. If the CFA results indicated an insufficient model fit, we proceeded with exploratory factor analysis (EFA) to uncover latent constructs within a dataset featuring a sample size under 300 and a non-normal distribution [35]. The suitability of the data for EFA was ascertained through Bartlett’s test for sphericity and the Kaiser–Meyer–Olkin (KMO) measure of sampling adequacy, where overall values ≥ 0.70 are desired [33].

For comparability with previous studies and robustness we performed the EFA using both generalized least squares (GLS) and DWLS. To aid in the interpretation of estimated factors, assuming moderate inter-factor correlations, promax oblique rotation (Kappa = 4) was used. In cases of ordinal data and non-normal distributed data, the DWLS method, which uses polychoric correlations, can produce more accurate estimates of the latent structures [36]. DWLS was therefore implemented using the R-package lavaan (v. 0.6.17) using the DWLS estimator.

In the present study, the cut-off for factor loadings was set at 0.32, with loadings above 0.40 considered significant, consistent with previous studies of the VSS [8, 11, 16, 18]. Typically, factor loadings above 0.40 are deemed significant, while a loading of 0.32 can be regarded as a minimum threshold [37]. Items that load at 0.32 or higher on two or more factors were considered cross-loading items.

##### Number of factors to retain

To mitigate bias, guidelines stress the importance of employing a variety of methods to ascertain the ideal quantity of internal characteristics [33,38]. The determination of the optimal number of factors to retain involved a multifaceted approach, utilizing four distinct parameters: the Kaiser criterion (eigenvalue greater than 1), the scree plot, Horn’s parallel analysis (HPA), and Velicer’s minimum average partial (MAP) method [39]. In addition, we considered prior studies suggesting that the instrument consists of two subscales: VSS–V and VSS–A.

#### Convergent validity

In assessing the convergent validity of our study, Spearman’s rank correlation coefficients were used to evaluate univariate relationships across three distinct hypotheses. The strength of these relationships was categorized as large (approximately ±0.50), moderate (approximately ±0.30), and weak (approximately ±0.10), in line with established academic standards [40]. For the VSS–SF acute version, we postulate as follows:

1. A large positive correlation between the total score of the VSS–SF and the total score of the Dizziness Handicap Inventory (DHI). This was based on the expected link between the frequency of vertigo-balance and autonomic-anxiety symptoms and the handicaps resulting from dizziness.
2. A moderate negative correlation between the VSS–V subscale with the balance test score, indicating an inverse relationship between these variables.
3. A moderate positive correlation between the VSS–V subscale with the average time taken to complete a clearly marked a 25-foot course (T25-FW).

For the standard VSS–SF, where the measurements are conducted after six weeks of vestibular rehabilitation, we postulate weaker correlations overall as we expect at least partial regression of the acute symptoms.

### Discriminant validity

Additionally, the scales’ ability to differentiate between ‘dizzy’ and ‘non-dizzy’ patients was examined using receiver operating characteristic (ROC) curves, with the area under the ROC curve (AUC) categorizing discriminative ability as acceptable (0.7–0.8), excellent (0.8–0.9), or outstanding (> 0.9).

95% confidence intervals for AUC were estimated using bootstrap resampling with 10000 replicates. For the VSS–SF acute version and its subscales used in the baseline studies, the study population (*n* = 86) was denoted as the ‘dizzy’ group. This group had undergone rigorous medical screening and fulfilled the criteria for AVS. The ‘non dizzy’ population comprised the control group with healthy adults (*n* = 54). In the application of the standard VSS–SF and its subscales following six weeks of vestibular rehabilitation, the term ‘dizzy’ was defined as meeting at least two of the following three criteria: a DHI score greater than 31, a performance within the lowest quartile on the T25-FW, and an inability to maintain balance for at least 30 s during the balance test.

### Reliability determination

#### External reliability

Test-retest reliability (reproducibility) was evaluated using intraclass correlation coefficients (ICC), where ICC values of 0.75–0.90 were considered good reliability and values above 0.90 were deemed excellent reliability [41].

#### Internal consistency reliability

The internal consistency of the VSS–SF, VSS–V and VSS–A subscales was evaluated, with Cronbach’s alpha values of 0.70 or higher considered satisfactory. Figure 1 presents a flowchart that outlines the statistical analyses conducted in the study.

### Software

Statistical analyses were performed using R 4.3.2 (2023, R-core team, Austria, Vienna) and IBM SPSS, version 28 (IBM Corp, Armonk, N.Y., USA). The Monte Carlo PCA for parallel analysis was conducted using a free software program developed by Watkins [42].

## Results

All participants in the study sample were symptomatic at inclusion and fulfilled the study criteria for AVS [21]. Out of the 100 participants who completed the questionnaires and tests, both at baseline and after six weeks of vestibular rehabilitation, including re-testing, 14 cases were excluded due to missing values, giving a total study sample of 86 (86%). No influential outliers or systematic differences were found between the total sample and the excluded cases. The descriptive information of the sample and control population is given in Table 3. No floor or ceiling effects were observed. The distribution between men and women was roughly equal in both the study sample (53.5% women) and the control group (51.9% women). The mean ages and age ranges of the two groups are nearly identical. The study sample had a mean age of 52.0 years (SD = 14.9), with ages ranging from 19 to 86 years, while the control group had a mean age of 52.1 years (SD = 17.6), with ages ranging from 18 to 94 years. A majority of the study sample were diagnosed with vestibular neuritis. The control group comprised of healthy individuals with no history of dizziness and vertigo.

**Table 3.** Demographic data and clinical status of the study sample.

Variable	Sample (*n* = 86)	Control, (*n* = 54)
Women, n (%)	46 (53.5)	28 (51.9)
Age; mean year (SD), min–max	52.0 (14.9) 19–86	52.1 (17.6) 18–94
Diagnosis; n (%)		
Vestibular neuritis	79 (91.9)	
Stroke	3 (3.5)	
Other	4 (4.7)	
Unspecified/unknown	3	
Laesio auris interna	1	
Educational level		
Elementary school (9 years), n (%)	8 (9.3)	
Upper secondary school (12 years), n (%)	30 (34.9)	
College/University (>12 years), n (%)	45 (52.3)	

SD: standard deviation.

The model fit indices for the CFA examining the assumed two-factor structure showed mixed results. The standardized root mean square residual (SRMR), comparative fit index value (CFI), and root mean square error of approximation (RMSEA) were 0.159, 0.955, and 0.130 (90% confidence interval: 0.108–0.152), respectively. As a result, exploratory factor analysis (EFA) was employed.

The 15 items in both the VSS–SF–SV, acute version used for baseline measurements, and the “standard” VSS–SF–SV used after six weeks of vestibular rehabilitation were subjected to EFA using IBM SPSS Statistics version 28. Prior to performing EFA, the suitability of data for factor analysis was assessed. Bartlett’s test for sphericity yielded significant results (*p* < 0.001), and the overall Kaiser-Meyer-Olkin measure was 0.74 for the acute version and 0.75 for the standard version, thereby confirming the suitability of the data for exploratory factor analysis.

**Figure 2.**
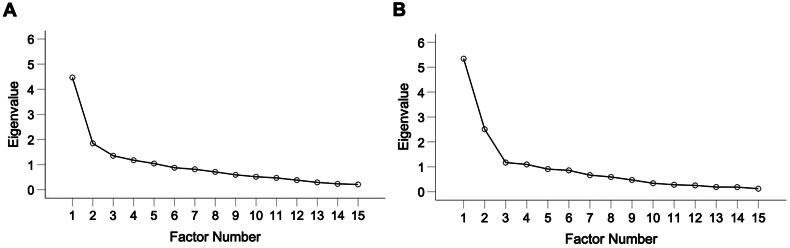
Scree plots of the EFA-analysis of A) acute version, and B) standard version of the VSS–SF.

In the acute version, EFA revealed the presence of five factors with eigenvalues exceeding 1, explaining 29.79%, 12.30%, 9.02%, 7.84%, and 6.96% of the variance respectively. An inspection of the scree plot (Figure 2(A)) revealed a clear break after the second factor. Using Catell’s (1966) scree test, it was decided that two factors should be retained for further investigation. This was further supported by the results of parallel analysis (Table 4), which showed only two factors with eigenvalues exceeding the corresponding criterion values for a randomly generated data matrix of the same size (15 variables x 86 respondents). In the case of DWLS, the two consecutive eigenvalues explained 30.1% and 16.8% of the variance, respectively.

**Table 4.** Results from parallel analysis vs actual eigenvalues from EFA.

Factor number	Criterion value parallel analysis	Actual eigenvalue, acute version (baseline)	Decision	Actual eigenvalue, standard version (6 weeks)	Decision
1	1,7882	4.468	Accept	5.344	Accept
2	1,5848	1.846	Accept	2.509	Accept
3	1,4523	1.353	Reject	1.173	Reject
4	1,3329	1.176	Reject	1.097	Reject
5	1,2250	1.044	Reject	0.909	Reject
6	1,1285	0.877	Reject	0.858	Reject
7	1,0369	0.815	Reject	0.667	Reject

Similarly for the standard version, the EFA revealed the presence of four factors with eigenvalues exceeding 1, explaining 35.63%, 16.73%, 7.82%, and 7.32% of the variance respectively. The scree plot (Figure 2(B)) revealed a small break between the second and third factor, and a larger break after the third factor. The results from the parallel analysis showed only two factors with eigenvalues larger than the corresponding criterion values from the Monte Carlo simulation, indicating only two factors to be retained for rotation. For DWLS, the two consecutive eigenvalues explained 39.7% and 22.9% of the variance, respectively.

The two-factor solution explained a total of 42.09% and 52.36% of the variance for the acute VSS–SF and standard VSS–SF, respectively. Using DWLS, the two-factor solution accounted for a cumulative variance of 47% for the acute VSS–SF and 62.6% for the standard VSS–SF. Significant factor loading values were set at 0.40 or higher, with modest loadings at 0.32 or more.

For the acute version, the promax rotation results (Table 5) showed that 11 items loaded clearly on two factors: seven on factor 1, corresponding to the VSS–V dimension, and four on factor 2, corresponding to the VSS–A dimension. Item 9 (shortness of breath), item 11 (excessive sweating), and item 12 (feeling faint) had modest loadings, while item 14 (chest pain) did not load significantly on either factor. Some items cross-loaded modestly on the opposite factor. Item 3 (nausea, vomiting) from VSS–V loaded on VSS–A, and item 12 from VSS–A loaded on VSS–V. Item 6 (dizziness, all day) weakly cross-loaded on factor 2. The factors correlated moderately (*r* = 0.46). Direct oblimin rotation (delta = 0) produced comparable results. DWLS analysis showed similar patterns, with 14 items loading significantly on either factor: eight items on factor 1, and six items on factor 2. Item 7 (headache, feeling pressure in the head) modestly loaded on factor 2, and items 3 and 12 showed consistent loading patterns with EFA.

**Table 5.** Item loadings in exploratory factor analysis (EFA) with a 2-factor solution.

	GLS^a^		DWLS^b^		GLS^a^		DWLS^b^	
	VSS–SF, acute version, at baseline				VSS–SF, standard version, at 6 weeks			
	F1	F2	F1	F2	F1	F2	F1	F2
**VSS–V**								
1. Vertigo (< 20 min)	**0.582**	0.087	**0.684**	0.002	**0.528**	−0.040	**0.611**	−0.162
3. Nausea, vomiting	0.135	**0.682**	0.248	**0**.**697**	**0.706**	0.069	**0.774**	0.115
4. Vertigo (> 20 min)	**0.484**	0.194	**0.629**	0.134	**0.770**	0.017	**0.874**	0.071
6. Dizziness (all day)	**0.506**	0.311	**0.680**	0.212	**0.671**	0.046	**0.822**	0.031
8. Difficulty to stand and walk	**0.720**	0.091	**0.754**	0.061	**0.675**	−0.010	**0.755**	−0.115
10. Unsteady (> 20 min)	**0.676**	0.026	**0.764**	0.029	**0.614**	0.030	**0.717**	0.087
13. Unsteady (< 20 min)	**0.952**	−0.275	**0.889**	−0.264	**0.780**	−0.215	**0.881**	−0.316
15. Dizziness (< 20 min)	**0.723**	−0.160	**0.806**	−0.286	**0.818**	−0.188	**0.892**	**−0.357**
**VSS–A**								
2. Hot or cold spells	−0.025	**0.748**	0.146	**0.682**	**0.327**	**0.512**	**0.365**	**0.583**
5. Heart pounding/fluttering	−0.157	**0.606**	−0.003	**0.608**	−0.067	**0.796**	0.078	**0.693**
7. Headache	−0.015	**0.429**	0.114	**0.376**	**0.563**	0.164	**0.514**	0.253
9. Short of breath	−0.127	0.275	−0.053	**0.494**	−0.002	**0.807**	0.016	**0.904**
11. Excessive sweating	0.087	**0.333**	0.158	**0.441**	**0.488**	**0.332**	**0.524**	**0.52**
12. Feeling faint	**0.344**	0.084	**0.616**	0.018	**0.331**	0.093	**0.375**	**0.331**
14. Chest pain	−0.199	0.145	−0.316	**0.636**	−0.111	**0.902**	−0.042	**0.995**

Factor 1 (F1): Vestibular-balance symptoms; Factor 2 (F2): Autonomic-anxiety symptoms.

^a^EFA using generalized least squares (GLS). Rotation method: promax with kaiser normalization. (Rotation converged in 3 iterations).

^b^EFA using polychoric correlation through robust diagonally weighted least squares (DWLS). Factor pattern coefficients of 0.32 or more, respectively, are displayed in bold.

For the standard version, promax rotation results showed 14 items loaded clearly on two factors: ten items on factor 1 and four items on factor 2. VSS–V items loaded adequately on the VSS–V dimension, with some items modestly cross-loading on VSS–A. Item 7 (headache) and item 11 (excessive sweating) in the VSS–A subscale loaded predominantly on factor 1, and item 12 (feeling faint) in the VSS–A subscale exhibited modest loading on factor 1 but did not load on factor 2. Item 3, which had an opposite loading pattern in the acute version, loaded adequately on VSS–V. The factors had a weak correlation (*r* = 0.24). Direct oblimin rotation (delta = 0) produced comparable results. DWLS analysis largely confirmed these findings, with 13 items loading adequately: nine on factor 1 and four on factor 2. Cross-loading and opposite loading patterns were consistent with EFA.

Cronbach’s alpha values, indicating internal consistency was 0.82 for VSS–SF, 0.85 for VSS–V, and 0.50 for VSS–A in the acute version, compared to 0.87 for VSS–SF, 0.86 for VSS–V, and 0.76 for VSS–A in the standard version.

Construct validity, as assessed using Spearman’s rank correlation coefficients (Table 6) showed a large positive correlation between VSS–SF and DHI (*r* = 0.68, *p* < 0.01), a moderate positive correlation between the VSS–V subscale and T25-FW (*r* = 0.46, *p* < 0.01), and a weak negative correlation between the VSS–V subscale and the balance test (r = −0.29, *p* < 0.01) for the acute (baseline) measurements.

**Table 6.** Spearman’s correlation of the scales with the comparators.

	VSS–SF	VSS–V	VSS–A	DHI	DHI–E	DHI–F	DHI–P	T25-FW	Balance
**Acute scales**									
VSS–SF		0.951^a^	0.665^a^	**0.680** ^a^	0.547^a^	0.670^a^	0.599^a^	0.472^a^	−0.319^a^
VSS–V	0.951^a^		0.419^a^	0.637^a^	0.528^a^	0.616^a^	0.552^a^	**0.456** ^a^	**−0.285** ^a^
VSS–A	0.665^a^	0.419^a^		0.477^a^	0.365^a^	0.488^a^	0.419^a^	0.232^b^	−0.252^b^
**Standard scales**									
VSS–SF		0.902^a^	0.780^a^	**0.544** ^a^	0.541^a^	0.495^a^	0.532^a^	0.017^c^	−0.325^a^
VSS–V	0.902^a^		0.496^a^	0.543^a^	0.479^a^	0.469^a^	0.583^a^	**0.047** ^c^	**−0.353** ^a^
VSS–A	0.780^a^	0.496^a^		0.364^a^	0.468^a^	0.333^a^	0.296^a^	−0.030^c^	−0.178d^c^

^a^Correlation is significant at the 0.01 level (2-tailed).

^b^Correlation is significant at the 0.05 level (2-tailed).

^c^Correlation is not significant, *p* > 0.05 (2-tailed).

Values highlighted in bold signify the correlations outlined in the previously stated hypotheses.

VSS–SF/VSS–V/VSS–A: Vertigo Symptom Scale-short form Total score/Vestibular-balance subscale/Autonomic-anxiety subscale.

DHI/DHI–E/DHI–F/DHI–P: Dizziness Handicap Inventory Total score/Emotional subscale/Functional subscale/Physical subscale.

Likewise, at six weeks, for the standard version of the VSS–SF and its subscales, the correlation coefficients indicated a strong positive correlation between the VSS–SF and the DHI (*r* = 0.54, *p* < 0.01) and a moderate negative correlation for the VSS–V subscale and the balance test (r = −0.33, *p* < 0.01). There was no correlation between the VSS–V subscale and the T25-FW (*r* = 0.05, *p* = 0.46).

### Test–retest reliability

The test-retest reliability after six weeks of vestibular rehabilitation was excellent for all scales with ICC values of 0.938, 0.932, and 0.925 for VSS–SF, VSS–V, and VSS–A respectively (Table 7).

**Table 7.** Test–retest of symptom scores at six weeks and reliability of the vertigo symptom scale-short as indicated by intraclass correlation coefficient (ICC) (*n* = 86).

Scale (range)	Test mean (SD)	Re-test mean (SD)	ICC (95% CI)
VSS–SF (0–60)	11.94 (10.12)	11.21 (10.11)	0.938^a^ (0.906, 0.959)
VSS–V (0–32)	8.10 (7.18)	7.69 (7.36)	0.932^a^ (0.898, 0.955)
VSS–A (0–28)	3.84 (4.51)	3.52 (4.13)	0.925^a^ (0.887, 0.951)

Two-way mixed effects model where people effects are random and measures effects are fixed. Absolute agreement.

^a^*p* < 0.001. SD: standard deviation, CI: confidence interval.

VSS–SF: Vertigo symptom scale – short form, VSS–V: Vertigo-balance sub-scale, VSS–A: Autonomic-anxiety sub-scale.

### Discriminant validity

**Figure 3.**
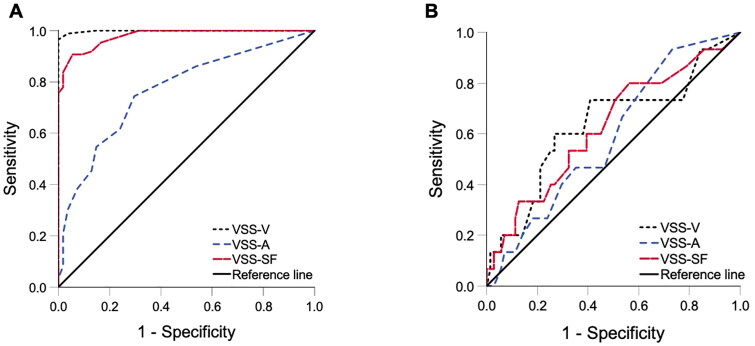
Receiver operating characteristic (ROC) curves. ROC curves demonstrating the discriminative ability of the VSS–SF and its subscales (VSS–V, VSS–A) between “dizzy” participants (*n* = 86) and “non dizzy” healthy controls (*n* = 54). Figure a shows the ROC curves at baseline (inclusion) using the acute VSS–SF and subscales. Figure B presents the ROC curves six weeks after vestibular rehabilitation using the standard VSS–SF and subscales. At six weeks, participants were classified as ‘dizzy’ if they met at least two out of three criteria: a DHI score greater than 31, placement in the worst quartile for T25-FW, or an inability to maintain balance for 30 s or more, resulting in 15 participants being classified as “dizzy”.

The ability to discriminate between ‘dizzy’ and ‘non dizzy’ participants in the acute phase was excellent for VSS–SF and the VSS–V subscale (AUC 0.978 and 0.998 respectively) and acceptable for the VSS–A subscale (AUC 0.766) as demonstrated by the area under the ROC curve (Figure 3(A), Table 8). Using the standard scales after six weeks of vestibular rehabilitation, the ability to discriminate between ‘dizzy’ and ‘non dizzy’ participants was diminished, although remained above 0.5, for all scales (AUC 0.627, 0.635, and 0.590 for VSS–SF, VSS–V, and VSS–A respectively) as shown by the area under the ROC curve (Figure 3(B), Table 8).

**Table 8.** The scales’ ability to discriminate between “dizzy” and “non dizzy” responders.

	Area under the curve (AUC), (95% CI)	
	Acute scales	Standard scales
VSS–SF	0.979, (0.957, 0.993)	0.627 (0.467, 0.779)
VSS–V	0.999 (0.995, 1.000)	0.635 (0.465, 0.794)
VSS–A	0.767 (0.685, 0.842)	0.590 (0.445, 0.731)

## Discussion

This study presents the first translation, cross-cultural adaptation, and validation of the Vertigo Symptom Scale – Short Form (VSS–SF) into Swedish. Additionally, it includes the first attempt to create a modified, acute version of the VSS–SF tailored to acute dizziness using a cohort with acute vestibular syndrome (AVS). The study adhered to a rigorous methodology for cross-cultural adaptation, resulting in the VSS–SF–SV. The procedural steps undertaken were predominantly aligned with established guidelines for translating and adapting PROMs [43,44]. Efforts to preserve the semantic and technical equivalence of the original English scale items were largely successful, despite some necessary linguistic modifications to fit the Swedish context. Notably, the English expression ‘feeling sick’ (item 3) was subsumed under the Swedish ‘illamående’ (nausea), and the term ‘swimmy’ (items 6 and 15) was omitted due to the lack of a direct equivalent. Furthermore, ‘heart pounding or fluttering’ (item 5) was translated as ‘hjärtklappning’, a term that closely aligns with the English ‘palpitation’ and encompasses both sensations effectively in Swedish. Pilot testing with a few acutely dizzy patients (*n* = 4) admitted to the hospital confirmed that these adjustments did not compromise the scale’s integrity, as patients reported clear understanding of the revised items and response categories. This careful translation and adaptation process is crucial for ensuring the reliability of the VSS–SF–SV in assessing dizziness and vertigo symptoms in Swedish-speaking cohorts.

Most existing studies on the VSS–SF involve participants with long-standing dizziness, exploring the scale’s application in the context of chronic symptoms. In contrast, our study focuses on the scale’s utility during the acute (duration 1–7 days) and subacute (duration 6 weeks) phase of vestibular symptoms, the former necessitating a modified, acute version of the VSS–SF to reflect a narrower temporal window. Instead of assessing symptoms over the past month, our acute version evaluates the frequency of symptoms within the past 24 h, acknowledging the immediacy and fluctuating nature of AVS. This adaptation opens the possibility of using the scale in clinical studies on acute vertigo.

The study’s balanced age and gender distributions help minimize confounding factors. The mean ages are nearly identical: 52.0 years for the study sample and 52.1 years for the control group, with overlapping age ranges. Gender distribution is also similar, with women comprising 53.5% of the study sample and 51.9% of the control group. This balance ensures that age and gender effects are evenly distributed, reducing the likelihood of these factors confounding the results.

Exploratory factor analysis (EFA) conducted on both the acute and standard versions of the VSS–SF confirmed a robust two-factor structure, corresponding well with the intended subscales for vertigo-balance (VSS–V) and autonomic-anxiety (VSS–A).

In the acute version, the two-factor solution explained 42.09% of the variance using Generalized Least Squares (GLS) and 47% using Diagonally Weighted Least Squares (DWLS). After rotation, 11 items clearly loaded on one of the two factors using GLS. Specifically, seven items (1, 4, 6, 8, 10, 13, 15) of the VSS–V subscale loaded on the VSS–V dimension with some weak cross-loadings, while four items (2, 3, 5, 7) loaded on the VSS–A dimension. Among the remaining items, item 9 (shortness of breath), item 11 (excessive sweating), and item 12 (feeling faint) showed modest loadings on VSS–A, while item 14 (chest pain) did not significantly load on either factor. Certain items exhibited loadings on the opposite factor, and some items had modest cross-loading. Specifically, item 3 (nausea, vomiting) from the VSS–V subscale loaded exclusively on the VSS–A dimension, suggesting it is primarily associated with autonomic anxiety symptoms in the acute stage of AVS. Item 12 (feeling faint) from the VSS–A subscale loaded on VSS–V, highlighting an overlap in symptom presentation, while item 6 (dizziness lasting all day) displayed weak cross-loading on VSS–A, indicating it may evoke both vertigo and autonomic anxiety responses. The moderate correlation between the two factors further supports the partial interrelation between vertigo-related and anxiety-related symptoms. The direct oblimin rotation corroborated these findings, confirming the robustness of our factor solution.

Factor analysis using DWLS also supported the bifactorial structure and showed similar loading patterns, but with a higher number of significant loadings on each factor: eight items on factor 1 (1, 4, 6, 8, 10, 12, 13, 15) and six items on factor 2 (2, 3, 5, 9, 11, 14). Item 7 (headache) had a modest loading on factor 2. Consistent with the GLS results, item 3 loaded on the VSS–A dimension and item 12 loaded on the VSS–V dimension. A notable difference with DWLS was that item 14, which did not load on any factor with GLS, clearly loaded on the VSS–A dimension with DWLS. The use of DWLS, which incorporates polychoric correlations, enhances the accuracy of factor loadings for ordinal data, providing a clearer distinction between factors. The consistency of the results using the two methods strengthens the validity of our factor structure.

For the standard version used post-rehabilitation, the total explained variance was higher at 52.36% with GLS and 62.6% with DWLS, possibly reflecting a more stable or developed state of symptom expression in patients. This version also showed a clear two-factor structure, with ten items loading on the VSS–V factor and three on the VSS–A factor with GLS. No opposite loading was observed for the items on the VSS–V subscale in the standard version, indicating a clearer distinction for these items as vertigo-balance symptoms develop and stabilize from the acute to the subacute stage of AVS. However, several items from the VSS–A subscale—item 2 (hot or cold spells), item 7 (headache, feeling pressure in the head), item 11 (excessive sweating), and item 12 (feeling faint, about to black out)—displayed opposite and cross-loading patterns on the VSS–V dimension. The weaker correlation between the two factors in the standard version suggests a more distinct separation between vertigo and anxiety symptoms compared to the acute version. Factor analysis using DWLS confirmed the exclusive loading of the VSS–V items on the VSS–V dimension, with modest cross-loading of item 15 (dizziness < 20 min) on the VSS–A dimension. The items from the VSS–A subscale showed similar loading patterns to the GLS analysis.

The loading issues seen with items 3 and 12 in the current study have also been reported in previous studies [8,11,16–18]. Item 3, which pertains to nausea and vomiting, frequently loads on both the VSS–V and VSS–A dimensions, suggesting it can be influenced by both vestibular and anxiety-related factors. Nausea and vomiting are related to autonomic nervous system disorders [45] and, from a physiological viewpoint, can be considered part of the autonomic-anxiety subscale. Yardley et al. (1999) intentionally retained this item in the vertigo and related symptoms (VER) subscale of the VSS long version because of its high face validity for the vertigo subscale, as balance disorders frequently provoke these symptoms [11], not least in the case of AVS where nausea and vomiting are common symptoms in the acute stage. As a result, the cross-loading and opposite loading patterns of such items can be expected, particularly for conditions like AVS, where vestibular and autonomic symptoms are often intertwined. In the current study, item 12 (feeling faint, about to black out) unexpectedly loaded on the VSS–V dimension in both the acute and standard version of the scale, with more modest loadings with GLS, and cross-loaded on the VSS–A dimension using DWLS in the standard version. This somewhat unexpected opposite loading pattern of this item has previously been observed in the Norwegian and Mexican translations of the VSS–SF and VSS scales, respectively. Additionally, item 7 (headache, feeling pressure in the head) and item 11 (excessive sweating) loaded as expected on the VSS–A dimension in the acute version but displayed significant cross-loading on the VSS–V dimension in the standard version, even showing stronger loading on VSS–V. The cross-loading of item 7 has also been observed in the Norwegian version of VSS–SF. The cross-loading and opposite loading patterns of the items in the VSS–A scale, particularly in the standard version, suggest a complex and dynamic interplay between vestibular-balance dysfunction and autonomic-anxiety components over time.

The internal consistency of the scales was robust, particularly for the VSS–V subscale across both versions, indicating a high degree of reliability in measuring vertigo-related symptoms. However, the autonomic-anxiety subscale presented a lower Cronbach’s alpha in the acute version, suggesting limited cohesion in capturing anxiety-related symptoms early in the disease process. These findings raise critical questions about the relevance and efficacy of the autonomic-anxiety subscale in the acute phase of AVS. However, the low internal consistency and moderate discriminative ability of the VSS–A subscale in the acute version may instead highlight potential issues with the questions’ ability to distinguish between anxiety-related and vertigo-related symptoms in the acute stage of AVS. The observed cross-loading of several items between the subscales may reflect the intertwined nature of these symptoms in acute vestibular conditions. Future refinements to the VSS–A subscale could focus on improving its specificity in measuring anxiety-related symptoms during the acute phase. It may be beneficial to investigate the development of these symptoms longitudinally to ascertain their trajectory and impact on patient outcomes.

The hypotheses regarding convergent validity were largely supported by the results. A large positive correlation between the total score of the VSS–SF and the DHI confirmed the expected relationship between vertigo-balance, autonomic-anxiety symptoms, and the handicaps associated with dizziness. A moderate negative correlation between the VSS–V subscale and the balance test score highlighted the expected inverse relationship, where greater symptom severity corresponded with poorer postural stability, consistent with previous findings [18]. Furthermore, the moderate positive correlation between the VSS–V subscale and the timed 25-foot walk (T25-FW) reinforced the scale’s ability to capture physical performance impairments related to vestibular dysfunction, in line with prior research [16].

These results demonstrate that the VSS–SF effectively assesses subjective symptom burden, while its correlations with objective physical function measures further support its validity as a comprehensive tool for evaluating vestibular disorders. The study’s findings validate the modified Swedish VSS–SF as a highly effective tool for early assessment of AVS, with excellent discriminative ability for vertigo-related symptoms. These results underscore the scale’s utility in clinical practice for rapid and accurate symptom assessment, which is essential for the timely management and treatment of AVS. A caveat is that the acutely dizzy participants reported using the VSS–SF acute version, while the non-dizzy participants used the standard version.

Future research could explore longitudinal patterns of autonomic and anxiety symptoms in AVS patients to gain more insight into how the VSS–SF’s autonomic-anxiety subscale develops over time. Additionally, further validation studies across different populations and settings could help adapt the scale for broader use, accommodating variations in symptom presentation and cultural contexts.

Nonetheless, the overall performance of the modified Swedish VSS–SF–SV, acute version particularly for assessing vertigo-related symptoms, was outstanding. The scale demonstrated excellent discriminative capability for distinguishing between ‘dizzy’ and ‘non-dizzy’ participants in the VSS–SF and VSS–V subscale. This underscores the scale’s effectiveness in clinical practice for swift and precise assessment of symptoms, which is crucial for the immediate management and treatment of AVS. After six weeks of vestibular rehabilitation, the ability of the VSS–SF–SV standard scales, and especially the VSS–A subscale, to differentiate between dizzy and non-dizzy participants decreased. Several theories might explain this finding:

First, the definition of ‘dizzy’/’non-dizzy’ was different at the 6-week follow-up compared to baseline. It is reasonable to assume that the healthy controls without any dizziness symptoms, comprising the ‘non-dizzy’ group at baseline, provide a starker contrast to the AVS group compared to the less dizzy rehabilitated AVS subjects constituting the ‘non-dizzy’ group at the 6-week follow-up.

Second, the effective resolution of acute symptoms through rehabilitation might contribute to the decline. As the immediate and more disruptive symptoms of vertigo diminish, the autonomic and anxiety symptoms, initially exacerbated by the vertigo, might also recede. This would likely result in lower scores on the VSS–SF in general, including the VSS–A subscale, reflecting an actual decrease in symptom severity.

Third, patients undergoing treatment for dizziness and vertigo could habituate to or psychologically adjust to their symptoms. This adaptation may lead to decreased acute anxiety and autonomic reactions, occurring alongside a rehabilitation-related reduction in vertigo symptoms. Over time, this could cause initially severe symptoms to be perceived as less significant, potentially lowering scores on the autonomic-anxiety subscales and impacting the discriminative ability of these measures.

These explanations underscore the need for ongoing evaluation and potential recalibration of the VSS–SF, especially the VSS–A subscale, to ensure it remains accurate and relevant in measuring symptoms across different stages of AVS recovery. By enhancing the scale’s sensitivity to these evolving patient experiences, clinicians can ensure it continues to serve as a robust tool for assessing the full spectrum of AVS symptoms throughout the treatment process.

## Conclusions

This study successfully translated, adapted, and validated the VSS–SF into Swedish (VSS–SF–SV), including the development of an acute version for early assessment of acute vestibular syndrome (AVS). The VSS–SF–SV demonstrated robust reliability and discriminative ability, particularly in assessing vertigo-related symptoms. The findings highlight the scale’s effectiveness in clinical practice for the timely management of AVS. However, the autonomic-anxiety subscale showed lower internal consistency in the acute version, suggesting a potential need for further refinement of the VSS–A items to improve its utility for symptom assessment in the acute stages of acute vertigo. Future research should focus on validating the scale across diverse populations and settings to enhance its applicability and sensitivity in measuring acute dizziness throughout different stages of recovery.

## Supplementary Material

Supplemental Material

## Data Availability

Data are available from the authors upon reasonable request. Contact solmaz.surano@umu.se or jonatan.salzer@umu.se.
