# Supplementary material for: The reliability and validity of the Swedish translation of the Vertigo Symptom Scale – short form in a cohort with acute vestibular syndrome
Source: Ann Med. 2025 Feb 10;57(1):2457517. doi: 10.1080/07853890.2025.2457517 (PMC11812103; doi:10.1080/07853890.2025.2457517)
Supplement: Supplemental Material [file IANN_A_2457517_SM7776.zip › Suppl/CONSORT_2010_Flow_Diagram_MS_Word (1).docx]

**CONSORT 2010 Flow Diagram**

## Enrollment

Included for analysis (n=86).

- Excluded from analysis (n=14) due to missing data/non-participation in either/or: VSS-SF, DHI, balance test, T25FW.
- Healthy controls (n=54) were recruited from a parallel study. See figure 1.

Completed VSS-SF (n=77)

- Withdrew from trial (did not wish to continue) (n=1)
- Missed follow-up at 6 weeks (n=6)
- Lost to follow-up (n=2)

## The first 100 participants to complete the VSS-SF questionnaire at 6 weeks were included into current study

Completed VSS-SF (n=84)

- Withdrew from trial (did not wish to continue) (n=3)
- Missed follow-up at 3 weeks (n=2)

Completed VSS-SF (n=80)

- Withdrew from trial (did not wish to continue) (n=1)
- Missed follow-up at 6 weeks (n=7)
- Lost to follow-up (n=1)

## 6-week follow-up

Completed Vertigo Symptom Scale-Short Form (VSS-SF) (n=89)

- Withdrew from trial (did not wish to continue) (n=4)
- Missed follow-up at 3 weeks (n=1)

## 3-week follow-up

Allocated to written instructions (n=89)

- Received allocated intervention (n= 89)

Allocated to online vestibular rehabilitation (n=94)

- Received allocated intervention (n= 94)

## Allocation

Randomized (n=183)

Excluded (n= 207)

- Not meeting inclusion criteria (n=103)
- Declined to participate (n=48)
- Other reasons (n=56)

Assessed for eligibility (n=390)
